# The Calcium Connection: Explaining Motor Neuron Vulnerability in ALS

**DOI:** 10.3390/cells15040322

**Published:** 2026-02-09

**Authors:** Tristan Dellazizzo Toth, Silvano Bond, Smita Saxena

**Affiliations:** 1 Department of Physical Medicine and Rehabilitation, University of Missouri, Columbia, MO 65212, USA; tdtbr@umsystem.edu (T.D.T.); sbnf8@umsystem.edu (S.B.); 2 Nextgen Precision Health, School of Medicine, University of Missouri, Columbia, MO 65211, USA

**Keywords:** ALS, calcium, motor neuron, neurodegeneration, selective vulnerability

## Abstract

**Highlights:**

**What are the main findings?**

- Calcium dysregulation plays a key role in ALS, acting on the cellular, synaptic, and network levels to drive disease progression.
- Calcium dysregulation is linked to metabolic dysfunction, ER stress, and aberrant neuron-glia interactions.

**What are the implications of the main findings?**

- Understanding the role of calcium dysregulation in ALS can elucidate the causes of the selective vulnerability of classes of motor neurons in ALS
- Targeting calcium dysregulation can lead to the development of new therapeutic interventions.

**Abstract:**

ALS is a severe neuromuscular disease classically characterized by the progressive loss of motor neurons, leading to incremental muscle weakness and eventually death. Current treatment options for ALS have proven to have limited effect, merely delaying the progression of symptoms and prolonging patient survival. This motor neuron subtype-related differential vulnerability has been linked to neuron excitability, metabolism, and protein aggregation. Calcium dysregulation, which serves as an important second messenger in neural signaling pathways, has been implicated in each of these mechanisms and represents a potential target for therapeutic intervention. Armed with cutting-edge tools for visualizing and recording calcium transients in vivo, ALS researchers have delved deeper into the role of calcium dysregulation in disease in recent years. Vulnerable motor neuron populations display an excess of calcium-permeable ion channels together with reduced expression of calcium-binding proteins, generating a cellular environment primed for excitotoxic stress. Loss of inhibitory synaptic input further heightens susceptibility to calcium overload. Paradoxically, some evidence suggests that elevated neuronal activity can exert neuroprotective effects, highlighting the complexity of activity-dependent calcium signaling in ALS. Additionally, ALS-related toxic protein accumulation disrupts calcium homeostasis, contributing to endoplasmic reticulum stress and mitochondrial dysfunction. Emerging data indicate that calcium dysregulation impairs neuron-glia communication, amplifying neuroinflammation and accelerating disease progression. This review aims to synthesize current evidence on how calcium imbalance contributes to motor neuron vulnerability and degeneration in ALS. By exploring the cellular, synaptic, and network-level mechanisms of calcium dysregulation in ALS, the review examines its interplay with mitochondrial and ER stress and explores its impact on neuron-glia interactions with the aim of synthesizing key mechanistic insights into the disease pathogenesis and therapeutic targets.

## 1. Introduction

The most common disease targeting motor neurons is Amyotrophic Lateral Sclerosis (ALS), with an estimated incidence of 1 to 2 people per 100,000 in the United States [1,2,3,4], with some regions such as the Midwestern states showing elevated levels closer to 8 people per 100,000 [5]. ALS affects both men and women, but the prevalence mildly skews male, and the typical age of onset is in late middle-age to older adults [5,6], although the rarer early-onset juvenile ALS is generally very aggressive [7]. The lifetime risk of developing ALS is approximately 1:350 for men and 1:400 for women, and the mean age of onset is 64 years [2,8,9]. Also known as Lou Gehrig’s disease, ALS carries a grim prognosis: progressive motor neuron degeneration and death in ALS results in median survival times of 3–5 years, with current treatments generally focused on managing symptoms and incrementally increasing lifespan [10,11,12]. Intriguingly, rates of progression can vary significantly with some patients showing a rapid onset of symptoms whereas others demonstrate a more gradual decline of motor functions [13].

### 1.1. ALS Etiology

ALS can be divided into two forms: Familial (fALS), which is inherited, and sporadic ALS (sALS), which arises with no known family history of the disease. The condition generally appears to occur randomly, without a clearly inherited genetic cause. The significant majority of ALS cases, approximately 90%, are sporadic, with only 10% being familial [4,14,15]. Environmental risk factors for disease include smoking, pesticide and heavy metal exposure [4]. Interestingly, when genetically screened, 10–20% of sporadic ALS patients are found to have mutations associated with fALS [5]. Overall, by 2024 over 40 genes have been causatively implicated in ALS, with mutations in these genes being linked to roughly two-thirds of all fALS cases and approximately 10–20% of cases of sporadic ALS, with several more being discovered in the decade since [16,17,18,19] and mutations in dozens more genes have since been implicated as being contributing risk factors [20]. Some of the most important genes found to be causative factors in developing ALS are *SOD1*, *TARDBP*, *FUS*, *VCP*, and *C9ORF72*, each accounting for at least 1% of ALS cases (Table 1) [17]. These genes have been linked to a variety of cellular functions and pathways. One of the most common causes of inherited ALS is mutations in the gene *SOD1,* which was also the first gene found to be associated with ALS [16,21]. Superoxide dismutase 1 (*SOD1*) encodes an antioxidant enzyme that breaks down toxic superoxide radicals into hydrogen peroxide and oxygen. Mutations in the *SOD1* gene account for approximately 12% of fALS and 1% of sporadic ALS [17,22]. TAR DNA-binding protein (*TARDBP*), which encodes the TDP-43 protein, regulates gene expression and acts as a transcription suppressor. Ubiquitinated TDP-43 has been found in neuronal aggregates that form in both ALS and frontotemporal dementia (FTD) [17,23]. *TARDBP* mutations account for about 4% of fALS cases and roughly 1% of sporadic ALS cases [17]. Mutations in the gene Fused in Sarcoma (*FUS)* account for approximately 4% of fALS cases and 1% of sporadic ALS. However, FUS mutations are the largest genetic source of juvenile ALS [7]. *FUS* which is another protein involved in transcriptional regulation and it is thought that its mechanism of action in ALS may be in a similar pathway to *TARDBP* [17,24]. Valosin-containing protein (*VCP*) mutations account for 1–2% of fALS cases [25]. Normal *VCP* function is necessary for the proper maturation of ubiquitin-containing autophagosomes and *VCP* mutations have also been linked to familial cases of Inclusion Body Myopathy, Paget disease, and FTD. Like *FUS*, the pathogenic action of mutant VCP is thought to be linked to *TARDBP*/TDP-43 [25]. *C9ORF72* mutations, particularly large hexanucleotide repeat expansions (GGGGCC) in the non-coding region, are by far the most common cause of familial ALS, accounting for at least 30–50% of familial ALS and they also cause a significant portion (7–10%) of sporadic ALS [26,27], while also being linked to FTD [17,28,29]. *C9ORF72* has been linked to a diverse range of cellular functions, including autophagy, membrane trafficking, and immune response [30]. *C9ORF72* hexanucleotide repeat expansions (HRE) have been linked to both haploinsufficiency and toxic gain of function driven by the aggregation of abnormal bidirectionally transcribed RNAs [28,31]. Consequently, targeting the *C9ORF72* HRE is seen as a prime therapeutic target. There is also evidence of interactions between heritable and environmental causes of ALS, with mutations in metallotionein (MT), transcription factor (MTF-1), and glutathione synthetase (GSS) genes being detected in several ALS patients and linked to impaired ability to breakdown and detoxify pesticides and heavy metals [32,33]. This constellation of genetic and non-genetic factors being linked to ALS suggests a disease etiology whereby the effect of environmental insults in driving disease manifestation is modulated by a patient’s underlying genetic susceptibility.

### 1.2. ALS Symptomology

**Figure 1:**
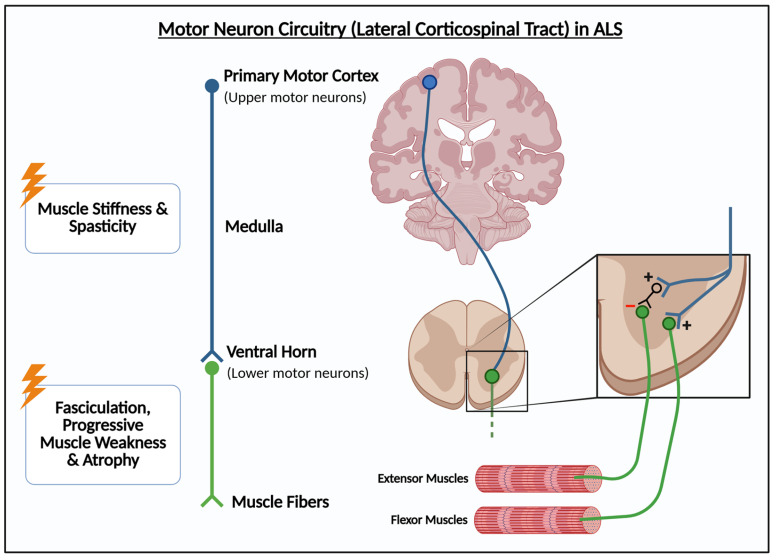
**Motor neuron circuitry and differential damage in ALS**. In ALS, degeneration of upper or lower motor neurons leads to a range of symptoms (**left**). Upper motor neurons stem from the primary motor cortex, decussate at the level of the medulla, and connect with lower motor neurons in the ventral horn of the spinal cord, which directly innervate muscle fibers (**center**). In the ventral horn, a combination of positive and negative synaptic inputs onto motor neurons constitutes the spinal circuitry, fine-tuning and coordinating flexor and extensor muscles to control movement and maintain stability (**right**). Created in https://BioRender.com.

ALS involves both upper and lower motor neuron dysfunction (Figure 1). Upper motor neurons are located in the motor cortex, and lower motor neurons are located in the brain stem and spinal cord. The upper motor neuron dysfunction in ALS causes muscle stiffness and spasticity, while the lower motor neuron dysfunction drives muscle fasciculations and then progressive muscle weakness, and atrophy as the neurons degenerate and they can no longer innervate their target muscles [10,11]. Roughly 10% of cases involve only upper or lower motor symptoms, referred to as primary lateral sclerosis and progressive spinal muscular atrophy, respectively [10]. However, both syndromes are still classified as subtypes of ALS due to autopsies demonstrating that there is almost always abnormalities in both upper and lower motor neurons regardless of symptoms [10]. Initially, these symptoms are peripheral, often starting with weakness in the hands or feet, but the dysfunction progressively spreads to most muscles, with facial muscles losing function, resulting in difficulty speaking, swallowing and, breathing [20]. Eventually, the disease progresses to functional paralysis, preventing patients from being able to walk. The cause of death is often due to paralysis spreading to the diaphragm and leading to respiratory failure [2,34,35]. ALS patients can also present with additional non-motor symptoms. ALS and frontotemporal dementia overlap in symptomology, with 50% of ALS patients showing FTD-like cognitive symptoms, such as cognitive decline and personality changes and, 30% of FTD patients also presenting with motor neuron impairments [20,36,37]. This overlap is understandable considering the shared genetic pathways between the two diseases.

Interestingly, there is a stereotypical progression of how and when motor units are affected in ALS (Table 2). The most vulnerable and severely affected are the large, Fast-twitch fatigable (FF) motor units [38]. These are controlled by the large diameter, myelinated, and fast-firing α-motor neurons, and each one contains several hundred muscle fibers and consequently, is the motor unit that is able to generate the most force. The contraction of FF muscle fibers is driven by anaerobic activity and hence is “fatigable” [39]. Next to show dysfunction are the moderately sized Fast Fatigue Resistant (FR) motor units, which use a combination of glycolytic and oxidative metabolism. Most resistant to dysfunction are the small, slow contracting, Slow fatigue-resistant (S) motor units, which rely exclusively on oxidative energy production [38,39].

## 2. Calcium-Driven Hyperexcitability and Excitotoxicity

Why do some motor neurons deteriorate faster than others in ALS? One prominent explanation points to differences in neuronal excitability, especially those driven by disrupted calcium regulation [14]. Calcium plays a key role in neuronal metabolism, signaling, and neurotransmitter release, and proper calcium homeostasis is essential for neural functioning [40,41,42]. Consequentially intracellular calcium concentration gradients are highly regulated [43]. Neurons tightly maintain the calcium gradient across their membranes, keeping intracellular concentrations on the order of 10,000 times lower than extracellular levels through employing membrane channels, chiefly ATPase pumps and Na^+^/Ca^2+^ exchangers (NCXs) [42,43]. Properly maintained calcium homeostasis is essential for correct metabolism, gene transcription, signaling and neuronal function. By contrast, calcium dysregulation has been linked to neuron dysfunction and degeneration [42,44,45].

A variety of changes in neuron structure, protein expression, and receptor composition in ALS have been linked to hyperexcitability (Table 3). Induced pluripotent stem cell (iPSC) derived motor neurons with *C9ORF72* mutations have elevated calcium release subsequent to depolarization, and after glutamate exposure, they have a delayed recovery to baseline calcium levels compared to controls [46]. They also have elevated spontaneous calcium transients [47]. It has also been found that AMPA receptors are more prevalent in vulnerable neurons in tissue from ALS patients, and, importantly, there are more AMPA receptors, with the iPSC-derived motor neurons expressing *C9ORF72* mutations there is more GluA1 expression and higher trafficking of AMPA receptors to synapses [48]. This can be ameliorated by CRISPR/Cas9-mediated correction of the *C9ORF72-HRE* [48]. Similarly, elevated levels of AMPA are present in iPSC-derived motor neurons with *FUS* mutations, and in iPSC-derived motor neurons with TDP-43 mutations have higher AMPA induced signal amplitudes [47]. Furthermore, the population of AMPA receptors expressed is also more permeable to calcium, which has been linked to reduced editing of the mRNA of the AMPA receptor subunit, GluA2, into its calcium impermeable conformation, with ALS patients showing reduced editing in the ventral horn [49,50,51]. This reduced editing of GluA2 mRNA, resulting in increased calcium permeability, has been linked to a failure of A-to-I editing at the q/r site by the enzyme *ADAR2* [52]. Mouse models lacking *ADAR2* showed a similar pattern of motor neuron degeneration as in ALS, with fast fatigable motor neurons degrading first while slow oculomotor neurons do not degrade until end stages of ALS [52]. Additionally, *C9ORF72* mutations lead to changes in dendritic arbor structure, spine morphology, and synapse and elevated activity of extrasynaptic GluN2B-containing *N*-methyl-d-aspartate (NMDA) receptors [53]. Similarly, iPSC-derived mutant TDP-43 motor neurons drove increased synapse formation and altered firing patterns [54]. Furthermore, vulnerable classes of motor neurons show reduced calcium buffering compared to less vulnerable ones [55,56,57]. The result of this is that vulnerable motor neurons in ALS are hyperexcitable and prone to calcium-induced excitotoxicity. Adding to the multiple studies involving in vitro research demonstrating maladaptive function of calcium-driven excitability changes, are additional lines of research in animals. Functional effects are demonstrated in vivo in *C9ORF72* expansion mouse lines showing neural disinhibition, abnormal activity during motor tasks and eventual cell death for pyramidal cells in the motor cortex [58]. Furthermore, there is evidence that vulnerable motor neurons in ALS also have a reduction in calcium-binding proteins, such as calbindin and parvalbumin, further increasing their risk of calcium overload and excitotoxicity [14,57,59,60,61]. Targeting calcium-induced excitotoxicity has been proposed as a target for future drug development [1]. Calcium channel blockers have been shown to ameliorate symptoms in certain ALS models but not others. The application of the voltage gated calcium channel blocker, lomerizine, proved to reduce cytoplasmic calcium buildup, toxic protein accumulation, mitochondrial dysfunction, and ultimately be neuroprotective in cultured motor neurons expressing mutant *SOD1*; however, it failed to produce similar effects in cultured motor neurons expressing mutant TDP-43 [62]. Other calcium channel targeting drugs currently being evaluated for ALS treatment include pimozide, which, in some cases, showed enhanced neuromuscular transmission in animal models and stabilization of function limited clinical trial [63], but failed to generate any improvement in others [64].

Additional evidence for the role of increased neuron excitability in ALS comes from motor neurons expressing mutations in the gene *FUS*, leading to upregulation of synaptic proteins, and an increase in synaptic vesicle release [65]. Furthermore, experiments involving injecting the serum of human ALS patients into mice resulted in elevated calcium levels in motor neurons and increased synaptic vesicles at axon terminals [66]. In addition to mechanisms of hyperexcitability in ALS driven by increased postsynaptic excitation, there is also some evidence for the role of disinhibition, with impaired inhibitory circuitry. There is mixed evidence for patients with ALS having dysregulated inhibitory input, with some studies showing a significant reduction in the inhibitory neurotransmitter, γ-aminobutyric acid (GABA) in the motor cortex [67], while others failed to find any difference compared to healthy controls [68]. Targeting the pathological motor neuron hyperexcitability and excitotoxicity in ALS is likely the mechanism behind the drug riluzole, which has the strongest evidence of any currently prescribed ALS drugs to extend the lifespan of ALS patients [69]. Riluzole acts to generally decrease the excitability of motor neurons through multiple mechanisms: it blocks voltage-gated sodium channels, inhibits voltage-gated calcium channels, and modulates potassium channels, ultimately inducing a reduction in glutamatergic transmission and preventing calcium overload [1].

## 3. Calcium-Driven Hyperexcitability as a Neuroprotective Mechanism

While calcium-driven excitotoxicity is well characterized as a late-stage maladaptive mechanism in ALS, there are also recent studies that suggest that the role of calcium and hyperexcitability in ALS pathology is more nuanced, sometimes being neuroprotective, particularly in the early stages of the disease (Table 4). When S- and F- type motor neurons were recorded early in development in a *SOD1* mouse model, counter to the hyperexcitability model, the vulnerable F-type motor neurons were not hyperexcitable, whereas S-type motor neurons were. Since S-type motor neurons are resistant to degeneration in ALS, this would suggest that hyperexcitability does not drive motor neuron degeneration [70]. Further research using *SOD1* mouse models has suggested that, at early stages, the ALS-induced hyperexcitability is not ubiquitous in vulnerable motor neurons [71]. Even at an early age (P8–P15), mutant *SOD1* aggregates are expressed globally but vulnerable neuron populations like trigeminal motor neurons do not show universal hyperexcitability. Instead, in this vulnerable population, there was a modification of neuron excitability that was based on their pre-existing firing thresholds. Firing thresholds were lowered in high threshold trigeminal motor neurons, but conversely, firing thresholds were increased in low threshold trigeminal motor neurons. These changes were not seen in ALS-resistant oculomotor neurons [71].

Combined with altered intrinsic excitability changes in ALS, there are modifications to the inhibitory synaptic inputs onto motor neurons. There is a progressive decrease in neuropeptide y expressing interneurons in the motor cortex of *SOD1* mice starting from 8 weeks old until end stages, and at end stages, a loss of calretinin expressing interneurons, which also show reduced dendritic arbor length, volume and reduced arbor complexity [72,73]. Additionally, in ALS, the reduction in both GABA receptor levels and its mRNA further points to inhibitory dysregulation [67,73,74]. When comparing glycinergic interneurons, which are the main inhibitory interneurons in the spinal cord, it was found that in mutant *SOD1* mice, the interneurons were less excitable with depolarized persistent inward currents, increased voltage thresholds for action potential firing and longer afterhyperpolarization duration [75]. Interestingly, as with motor neurons, there is a stereotypical order of progressive dysfunction and degeneration of spinal interneurons. Ventral interneurons showed the most pervasive decline, while interneurons in lamina 7–8 were the most preserved [75]. Indeed, spinal interneurons as a whole showed earlier degeneration kinetics than spinal motor neurons in a *SOD1* model [76]. This suggests that disinhibition plays a key role in ALS. This is through the attenuation of inhibitory interneuron input, particularly on interneurons that synapse onto vulnerable fast-twitch motor neurons, especially early in disease [76]. This disinhibition drives changes in motor neuron intrinsic excitability, particularly selectively lowering baseline firing thresholds in vulnerable motor neuron populations. This frames ALS as a disease of circuit dysfunction, in which the motor neuron degeneration is the end-symptom of an imbalance in excitatory and inhibitory synaptic inputs. This also suggests that drugs like riluzole, which universally depress central nervous system excitability, could exacerbate this dysfunction in inhibitory interneurons, even as they are directly targeting motor neuron excitability. This could be the reason why riluzole treatment generally only extends ALS patient survival by timespans measured in months rather than years [69]. This suggests that drug treatments that target calcium levels in a more cell-type-specified manner could yield better clinical results. Together, the results of these studies suggest that the interaction among calcium, altered motor neuron excitability, cell death and disease pathology is not just a straightforward, linear interaction. Instead, a complex interaction occurs in which subgroups of vulnerable and less vulnerable neurons shift their activity thresholds based on their preexisting properties and shifts may be either compensatory or maladaptive.

Motor neuron excitability as a neuroprotective mechanism provides a mechanism as to why different classes of motor neurons are generally more or less vulnerable and why there is a stereotypical progression of dysfunction in ALS. On one extreme, the large, fast firing motor neurons are the most vulnerable and first affected because they are the least excitable in line with Henneman’s size principle [77], whereas the smaller, S motor neurons are generally spared in ALS until the end stages because they have a low threshold for excitation [78]. Interestingly, recent research has found that elevated calcium levels also appear to have a feedback effect on the expression of key ALS-associated proteins such as *C9ORF72* and *SOD1*, reducing their expression through driving the activation of calpains and caspases [79]. Toxic accumulation of the ALS-associated protein TDP-43, which occurs in 95% of ALS cases [27], could also be reversed by elevating intracellular calcium levels in drosophila sensory neurons [80]. Additionally, it has been found that the Calcium/calmodulin dependent phosphatase, calcineurin, which is activated in response to increased intracellular calcium levels acts to directly dephosphorylate TDP-43 and this prevents toxic accumulation [81,82,83]. In animal models of ALS, calcineurin is depleted, while calcineurin overexpression protects vulnerable neurons from neurodegeneration [81]. Furthermore, there is evidence that calcium channel agonists can act to prevent motor function decline in animal models [84]. Together, these results demonstrate that calcium-driven increases in neuron excitability can play a neuroprotective role in ALS.

So, how can calcium act as both a driver of hyperexcitability and excitotoxicity while also being neuroprotective in ALS? The answer to this is that the impact of calcium-driven excitability changes in ALS is dynamic over the course of the disease. Initially, calcium-driven changes in motor neuron excitability observed early in ALS progression are not a simple maladaptive change but rather an early compensatory mechanism, which, for a time, acts to counter pathological dysfunction. In later stages, when the compensation mechanisms are eventually overwhelmed, the role of calcium switches to being primarily excitotoxic. These differing consequences of calcium-driven excitability changes at different temporal stages of ALS could also impact why studies using models with similar genetic backgrounds (e.g., *SOD1*) could yield seemingly conflicting results. However, it is also worth noting that the use of different model systems, primarily in vitro human cells or in vivo rodent models, in different studies, which have different benefits and drawbacks (summarized in Table 5) in different studies, could have an impact. Importantly, the time-course dependent role of calcium-driven excitability changes in ALS has implications for potential therapeutic interventions. In the early stages, raising intracellular calcium levels represents a potential therapeutic target for protecting vulnerable motor neuron populations, whereas in later stages where excitotoxicity is prevalent, treatments that prevent calcium overload in neurons would likely be effective.

## 4. Calcium, Metabolic Function, and Endoplasmic Reticulum Stress

Recent research has also shaped our understanding of the effects of changes in excitability in ALS pathology. In particular, it has been found that motor neuron excitability can be protective in ALS [85]. In a *SOD1* mouse model, the most vulnerable population of motor neurons, fast fatigable motor neurons, which have low excitability, show neuroprotection by enhancing their excitability, activating mTOR, and reversing misfolded *SOD1* accumulation [85]. Additionally, in the presymptomatic phase, inhibiting the excitability of alpha motor neurons results in the induction of ALS-like pathology, while increasing motor neuron excitability slows motor decline and neuron denervation [85].

The accumulation of misfolded *SOD1* is known to drive ER stress in ALS, and this is linked to changes in key calcium-binding proteins [86,87,88,89]. In ALS patients, misfolded *SOD1* is found to colocalized to the ER [86,87]. Furthermore, research shows that in ALS mouse models, motor neurons that are selectively vulnerable, in particular Fast Fatigable motor neurons, progressively activate an ER stress response [89,90]. The mechanism driving the activation of the ER stress response and subsequent selective neuron death is a reduction in the level of a calcium-binding ER chaperone, calreticulin [89]. Reduced calreticulin, which is only observed in vulnerable motor neurons, drives the activation of the Fas/NO pathway and triggers cell death and muscle denervation [89]. As toxic proteins continue to build in the ER, the Unfolded Protein Response (UPR) is triggered, which acts to counter this by increasing transcriptions of ER-resident chaperones, reducing the ER protein load by down-regulating protein synthesis more broadly, and driving increased degradation of misfolded proteins [91]. However, when the ability of the UPR to counteract the buildup of misfolded proteins is overwhelmed, it subsequently leads to the activation of apoptotic pathways [91]. Consequently, treatment with an ER stress-protective agent, salubrinal, slowed disease progression and ameliorated symptoms [90]. Similarly, intracerebroventricular injection of cerebral dopamine neurotrophic factor (CDNF) was shown to reduce ER stress and halt disease progression in both *SOD1* and *TDP43* rodent ALS models [92]. However, recent research suggests that ER stress could be a protective compensatory mechanism to ALS disease pathology, rather than a maladaptive mechanism [93]. Acetylcholine signaling blockade in a *SOD1* model was effective at reducing ER stress but also increased misfolded *SOD1* levels and prevented mTOR activation in vulnerable motor neurons [85].

**Figure 2:**
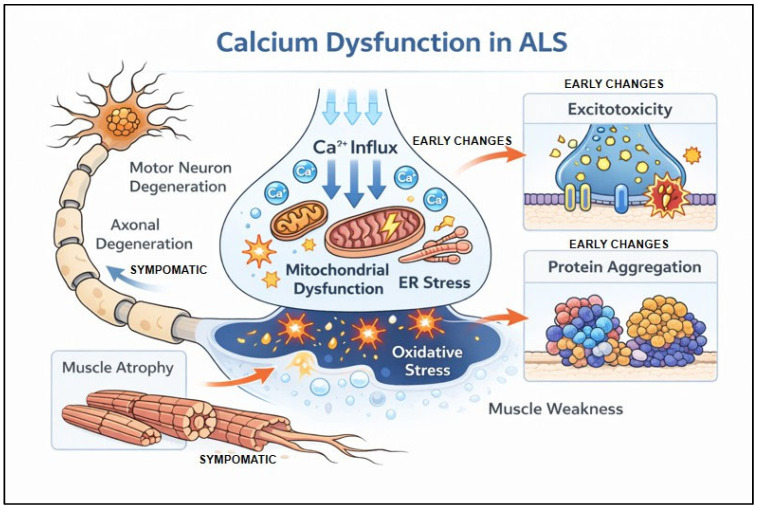
**Calcium-mediated neuronal damage in ALS.** Schematic depicting how elevated intracellular calcium impairs mitochondrial and ER function, promoting protein aggregation, and resulting in excitotoxicity, circuit alterations, and muscle atrophy. Moreover, excessive intracellular calcium in motor neurons triggers oxidative stress and organelle dysfunction. Created in https://BioRender.com, and additional modifications were done using Adobe Photoshop 2025.

Recently, more research has focused on the roles of ER-mitochondrial interaction, dysfunction, calcium buffering in ALS (Figure 2). The precise control of intracellular calcium homeostasis through the ER-mitochondria calcium cycle plays an important role in ALS pathology. Mitochondria and the ER act as intracellular calcium buffers, rapidly sequestering large influxes of cytosolic calcium [94,95]. In the ER, calcium release is controlled by ryanodine receptors and inositol 1,4,5-triphosphate receptor-gated channels and uptake is facilitated through the sarco/endoplasmic reticulum Ca^2+^ ATPase [91,96,97]. In the mitochondria, calcium is taken up via the mitochondrial uniporter and extruded using the Na^+^/Ca^2+^ and 2H^+^/Ca^2+^ exchangers [91,98,99]. The ER and mitochondria exchange calcium to maintain homeostasis through a process termed the ER–mitochondria Ca^2+^cycle [55,91]. In ALS as well as other neurodegenerative diseases like Frontotemporal dementia, Parkinson’s, and Alzheimer’s, there is evidence of disruption in these different functions [46,91,94,100]. Vulnerable motor neuron populations in *SOD1-G93A* mutant mice have reduced cytosolic calcium clearance, perturbed mitochondrial calcium extrusion, and excessive ER calcium uptake by the sarco-/endoplasmic reticulum Ca^2+^ ATPase, and targeting these impaired calcium-control mechanisms was neuroprotective [95,101]. Similarly, in *C9ORF72* iPSC-derived motor neurons, there are elevated mitochondrial calcium levels and impaired mitochondrial function, both of which can be reversed by treatment with Talineuren, a nanoliposome-based formulation of GM1 ganglioside [100].

The interplay between calcium, metabolic function, and ER stress in ALS is dynamic over the course of the disease. In mitochondria, increased calcium uptake due to rising cytosolic calcium levels in vulnerable neurons has the potential to initially augment mitochondrial respiration, preventing excitotoxic injury. This is supported by evidence that in ALS patients, there are increased neuronal calcium levels, an increase in mitochondrial volume, and the formation of mitochondrial conglomerates in the lumbar spinal cord [91]. However, eventually this leads to mitochondrial overload and calcium dysregulation [94]. It is known that ER stress first arises quite early in ALS in the prodromal stage, preceding metabolic dysregulation and neuronal death [102]. In studies using patient-derived *C9ORF72* motor neurons, it was discovered that this early disease ER stress was a mediated adaptive response, particularly through driving metabolic activity [103]. In the early stages of disease progression, there is an increased *GRP75* expression, which drives enhanced ER/mitochondria association. This is key to boosting mitochondrial function, and allows the motor neurons to successfully compensate in early disease stages for dysfunction and ultimately sustain sufficient metabolic outputs [103]. As the disease progresses, *GRP75* levels drop when the UPR is triggered. Subsequently, there arises mitochondrial dysfunction, polyGA aggregates, and mitochondria have failures in their ability for calcium reuptake and can no longer act as buffers [26,46,103]. This is a function that is especially important in an ALS context, as affected motor neurons already have a deficit of functional calcium buffering proteins like parvalbumin, calbindin and calreticulin [14,59,72,73]. If, however, *GRP75* expression is sustained at a high level, ER stress is ameliorated, toxic misfolded protein expression is prevented, and proper mitochondrial calcium buffering function can be maintained [103]. This suggests that the ER stress pathway is a key response of cells maintain homeostasis early in disease, and that its dysregulation and the activation of the UPR lead to disease progression. Drugs such as kaempferol, which modulate *GRP75* expression, act to correct the aberrant mitochondrial calcium reuptake and ameliorate ER stress and the activation of the UPR, have proven effective in preventing the progression of both motor dysfunction and neuron degeneration in *C9ORF72* mouse models and *C9ORF72*-ALS human patient-derived motor neurons [46].

## 5. Calcium and Toxic Protein Aggregation in ALS

Mutations in *SOD1* cause toxic protein aggregates, ER stress, and activation of the Fas pathway, which in turn drives motor neuron death [104]. Toxic *SOD1* is also linked to motor neuron metabolism, with aggregates also prevalent in the mitochondria, and they drive aberrant mitochondrial function even before the onset of motor symptoms [105]. Additionally, expression of mutant *SOD1* in mouse cell lines results in aberrant Golgi fragmentation, reduced mitochondrial membrane potential, increased oxidative stress, and an elevated basal calcium level [106]. Misfolded *SOD1* also inhibits the ATPase activity of Na^+^/K^+^ATPase-α3, a key component of the ability of neurons to maintain an electrochemical gradient [107]. Another toxic gain-of-function protein aggregation that has been linked to ALS pathology via calcium dysregulation is *AnexinA11*, which encodes a calcium-dependent phospholipid-binding protein. Mutations in *AnexinA11* have been found to occur in some ALS patients and have caused alterations in calcium homeostasis and stress granule disassembly [108].

## 6. Calcium Dysregulation in Glial Cells in ALS

Similar to neurons, calcium signaling in glia serves as key regulator of function and homeostasis, facilitating their roles in neuronal support, synaptic modulation, and immune response. Consequently, mutations in several previously identified ALS-causative genes have also been implicated in glial cell calcium dysfunction. The toxic gain-of-function of mutant *SOD1* not only affects motor neurons directly but also affects them indirectly through modifying interactions with glial cells. Microglia expressing mutant *SOD1* are known to attack motor neuron synapses, drive axonal damage, and cell death, in parallel to also indirectly driving hyperexcitability in motor neurons through activating other immune cells [109,110,111,112]. In addition, the selective blocking of the pro-inflammatory-linked KCa3.1 channel in *SOD1-G93A* mouse microglia resulted in significant delays in symptom progression and partially rescued fundamental microglial functions, such as surveillance activity and microglia-neuron crosstalk [113,114]. The resulting amelioration of muscle denervation in consequence to blocking KCa3.1 suggests that targeting this channel could serve as a valuable therapeutic target for the treatment of patients with *SOD1*-driven ALS. Additionally, mutations in the *FUS* gene have been shown to critically impair chemoreceptor-activated calcium signaling in human iPSC-derived microglia [115]. As microglial calcium transients are linked to the modulation of physiological and pathological neuronal activity, these impairments likely affect microglial responses to damage, possibly leading to an increased risk for neurodegeneration.

Likewise, for astrocytes, the intracellular signaling is also highly calcium-dependent. Many ALS-linked mutations, including those affecting *SOD1*, *TARDBP*, and *FUS* genes, are associated with disturbances in astrocyte ER and mitochondrial function. Indeed, they are related to impaired store-operated calcium entry (SOCE) via Orai1 and STIM1, which promotes ATP release and the production of pro-inflammatory cytokines [116,117,118]. Importantly, in vivo evidence in *SOD1-G93A* astrocytes identified a SOCE-dependent mechanism leading to calcium dysregulation, which contributes to ALS pathogenesis [118]. Early studies highlighted a link between a mutant *SOD1* and persistent reduction in membrane resistance, as well as a rise in intracellular calcium activity [119]. Subsequent studies in murine *SOD1-G93A* primary astrocytes provided evidence of increased SOCE, lower expression of sarco-endoplasmic reticulum Ca^2+^ ATPase, as well as lower resting calcium concentration [120]. Additionally, the presence of TDP-43 inclusions significantly reduced stimulation-induced cAMP and calcium increases in cultured murine astrocytes [121]. Media from astrocytes that express mutant *SOD1* are shown to induce increased sodium inward currents, repetitive firing, and intracellular calcium transients in exposed wildtype spinal cultures, leading to motor neuron cell death [122,123]. Treatment of these conditioned neurons with riluzole was effective at reducing the elevated calcium transients and preventing neuron death [122]. Research provides causal evidence of astrocyte dysregulation and enhanced glutamate release at neighboring synapses in a mouse *SOD1* model, further demonstrating their impact on motor neuron degeneration [124]. In addition, satellite glia also are dysfunctional and lose their ability to clear excess glutamate, leading to elevated glutamate levels around motor neurons and, consequently, excessive calcium influx, which could amplify excitotoxic mechanisms in ALS [93]. To date, no definitive evidence firmly establishes a causal temporal order between glial and neural calcium dysregulation. However, early-phase microglial activation has been previously described, with increasing evidence defining an initial “neuroprotective” role for microglia and astrocytes in early disease stages, followed by a switch to a “neurotoxic” state that might accelerate motor neuron degeneration [125,126]. While current evidence suggests a co-evolution between motor neurons and glial cells, these findings remain preliminary and require more in-depth studies. Currently, the study of the impact of calcium activity in glial cells and ALS pathology is still an emerging field of study. However, recent research linking aberrant calcium activity in glial cells to neural decline and loss of function provides avenues for new therapeutic targets.

## 7. Tools for Analyzing Calcium in Motor Neurons

**Figure 3:**
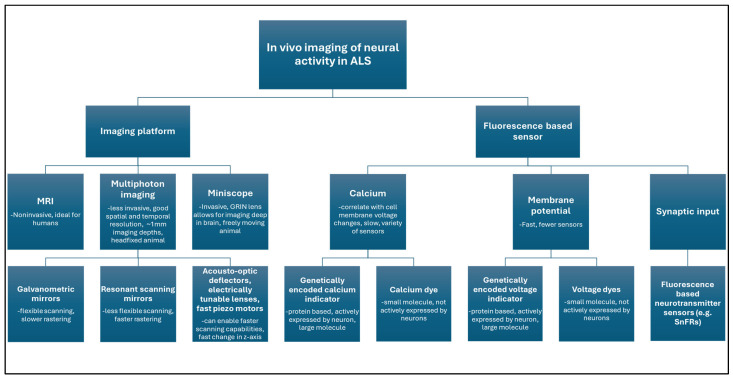
**In vivo imaging technologies.** Imaging technologies and fluorescence-based sensors that have been generated and can be applied to investigating neural activity in vivo, ALS, and ALS animal models.

One major challenge facing ALS researchers analyzing the functional effects of the disease on neural circuits and activity is being capable of recording that activity. Neural calcium transients and neurotransmitter release are by their nature, dynamic processes. Thus, in many cases traditional histology or immunofluorescence imaging of fixed tissue is insufficient for accurately capturing pathologies in these processes. Consequently, a host of tools have been developed to enable imaging of activity in live neural tissue. In vitro imaging of live neural tissue has been successfully achieved in cultured neurons using a variety of both commercial and customized fluorescence-based imaging platforms, including fast wide-field or confocal imaging [127]. In contrast, in vivo imaging of neural activity presents several challenges that researchers must overcome if they are to unlock the interactions between activity and disease (Figure 3).

### 7.1. In Vivo Imaging Platforms

Standard single-photon fluorescence imaging has a limited imaging depth of merely a few hundred microns, which is insufficient to sample calcium activity in all but the most superficial layers of neurons [128,129]. Consequently, two-photon imaging has been employed, which leverages the reduced scattering properties of near-infrared wavelengths, allowing for imaging at millimeter depths [130,131]. Recently for deeper brain imaging, three-photon imaging has been employed, which, when combined with adaptive optics, can allow for imaging depths of several millimeters [132,133,134]. Alternatively, for deep-layer in vivo imaging, Miniscopes containing embedded GRIN lenses can be employed, which have the advantage of being light-weight and small, enabling the recording of calcium transients in freely moving animals, albeit at the cost of reduced resolution and invasiveness [135,136]. Miniscope systems have proven invaluable for interrogating neural circuit function in neurodegenerative diseases, including ALS, with it being particularly useful for longitudinal studies, where long-term activity recording is essential [137,138]. Very recently, two-photon imaging and Miniscope-like head-mounted systems have been combined into a single platform that combines the depth and rapid volumetric imaging of two-photon with the long-term recording capabilities of free behavior of miniscopes [139].

Another key imaging challenge to overcome is the need to sample at rates fast enough to record calcium transients with sufficient temporal resolution. High temporal resolution is particularly essential for in vivo imaging in ALS models so that dysregulation in calcium transients can be accurately recorded and characterized. In a typical in vivo multi-photon imaging system, the scanning laser is controlled in the x and y- axis by either a pair of galvanometric mirrors, which have the advantage of scan space flexibility at the cost of slower maximum scanning speed over a large field of view or alternatively, a set of resonant scanning mirrors, which allow for faster scanning rates at the cost of increased distortions and not being able to flexibly dictate the scan area or mirror dwell time [140,141]. When fast sampling through 3D volumes is needed, various methods are employed, such as using fast piezo motors, acousto-optics, and electrically tunable lenses, often in combination to be able to achieve fast sample rates in all three axis [142,143,144].

### 7.2. Fluorescence-Based Constructs

Imaging platforms that can sample neural tissue in in vivo ALS models with sufficient spatial and temporal resolution are of limited use without the fluorescent constructs capable of reporting activity. To that end, there is a wide array of tools available for interrogating the link between disease state and calcium activity. Rapid inward transients in intracellular calcium correlate strongly with the depolarization at the cell membrane associated with excitatory synaptic input and action potentials [42,145,146]. Consequently, calcium-sensitive fluorescent dyes and genetically encoded calcium indicators (GECIs) have been developed and refined, with a highly utilized tool being the GCaMP series of fluorescent calcium sensors, with recent iterations demonstrating unparalleled sensitivity and dynamic range [146,147,148,149]. Also adding to the usefulness of these calcium sensors, like GCaMP, as an indicator of neural activity, is that the fluorescence-based recordings of both synaptic and action potential-mediated calcium influxes are significantly slower than the underlying voltage change across the membrane due to their calcium-binding kinetics [145,146,149,150]. This has greatly reduced the temporal resolution requirements and made calcium sensors much more accessible tools then fluorescence based voltage sensors, although developing deconvolution tools is often necessary to process out noise and extract the underlying spike dynamics [143,144,151,152].

Fluorescence-based calcium sensors have been instrumental in unraveling calcium dynamics in ALS. Combining two-photon imaging and AAV-driven GCaMP expression in awake, freely moving TDP-43 mouse model of ALS has further elucidated the role of microglia in disease-driven neuronal hyperexcitability, through them directly interacting with and modulating neuronal synapses in a disease model [153]. Further in vivo experiments analyzing disease-associated neuronal excitability in ALS utilizing 2-photon imaging and GCaMP, have been performed to link aberrant TDP-43 protein aggregation and attenuated calcium transients in spinal motor neurons [154]. Fluorescence-based calcium sensors have also been combined with miniscopes to elucidate neural calcium dynamics in situations where free movement and long-term recording of the research animal, typically a mouse, is essential [61]. Calcium activity in mice where TDP-43 depletion were monitored for several months and it was discovered that in these mice, this depletion resulted in hyperactive calcium activity, rapid activity decline, and eventual neuron death [138]. The combination of fluorescence-based calcium sensors and imaging platforms like two-photon and miniscope unlocks valuable capabilities for interrogating neural activity in in vivo animal models. Fluorescence-based sensors like GCaMP, which are valuable tools for directly recording calcium transients in post-synaptic neurons, have recently been complemented by constructs designed to record the synaptic inputs themselves that drive these calcium constructs [155,156,157]. This is important because emerging evidence suggests that patterned inputs may drive non-linear signal summation and calcium flux [158,159]. Consequently, fluorescence-based constructs that bind to specific neurotransmitters have been developed [155]. These constructs, termed “SnFRs”, have been applied to investigating perturbations of synaptic inputs into ALS models [160]. Complementing fluorescence-based calcium and neurotransmitter binding constructs is the application of channelrhodopsins (ChRs). When expressed in neurons, these photoactivatable ion channels can be used as molecular switches, turning activity on or off in particular neurons by applying a specific wavelength of light [161,162]. Optogenetic systems have enabled the generation of novel animal models of ALS, which allow scientists to precisely and reversibly control disease onset [163]. Not only does this optogenetic control provide a valuable tool for investigating disease circuits, but optogenetics is also being explored as a potential mechanism for treatment in ALS models, with ChR activation being used to drive the restoration of innervation of severely affected skeletal muscles in *SOD1* mice [164]. Together, these tools enable the recording of both neural input and the resulting calcium spike output in ALS models, and they also allow manipulation and interrogation of these pathways, opening up new and exciting potential therapeutic options.

### 7.3. Imaging Calcium in Human Patients

Fluorescence-based laser scanning microscopes have proven to be an excellent tool for interrogating ALS mechanisms in animal models; however, due to the inherent invasiveness of these technologies, their application in human ALS patients is limited. As an alternative, non-invasive imaging technologies have been applied. Through the recent application of ultra-high MRI imaging capable of generating submillimeter resolution in vivo images, it was possible to assess the levels of calcium markers in specific cortical layers [165,166,167]. This led to the finding that in ALS patients, in M1, there is significantly increased calcium in layer 5a and the superficial layer, and that calcium hotspots were predictive of demyelination [166]. Through utilizing emergent technologies in MRI, ALS researchers and clinicians now have access to imaging calcium dynamics in live patients, opening the door for analyzing the role of calcium in the disease in a direct manner.

## 8. Conclusions

ALS is a devastating, incurable disease that decimates motor neuron populations. However, the destruction is not random, and the order in which motor neuron populations are targeted offers clues into the disease mechanism. A key driver of why some motor neuron populations are resistant to degeneration has been proven to be their activity and calcium dynamics. An impressive array of optical imaging technologies and genetic constructs has been applied to be able to further investigate the link between aberrant activity patterns and ALS pathology. On the surface, ALS disease progression appears to be driven by a relatively simple mechanism of hyperexcitability, and excitotoxicity. However, recent research has revealed that ALS pathology is governed by aberrant activity and dysfunctional calcium flux and buffering. There is a consequent complex interplay of excitation, disinhibition, hypoactivity, toxic protein aggregation, and neuron-glia interactions, leading to vulnerability patterns that are characteristic of ALS. Excitability and calcium also play a key role in ER stress and mitochondrial function in affected motor neurons. Our emerging understanding of the dynamic role of calcium in ALS pathology opens the door to developing new potential therapeutic treatments that could selectively target specific dysfunctions across different cellular populations (Table 6). This offers hope for a new class of treatments for a disease that has thus far proven incurable.

## Figures and Tables

**Table 1:** Key causative genes in ALS.

Gene	% Accounting for fALS	% Accounting for sALS	Protein Function	Pathogenic Function in ALS
*SOD1*	12	1	antioxidant/superoxide metabolism	toxic protein aggregation, calcium dysregulation, ER stress, metabolic dysfunction, microglia and astrocyte dysfunction
*TARDBP*	4	1	transcription regulation	toxic protein aggregation, calcium dysregulation, aberrant synapses, ER stress, astrocyte dysfunction
*FUS*	4	1	transcription regulation	linked to TARDBP dysfunction, calcium dysregulation, aberrant synapses, microglia dysfunction, astrocyte dysfunction
*VCP*	1–2	1	autophagosome function	linked to TARDBP dysfunction, metabolic dysfunction
*C9ORF72*	30–50	7–10	autophagy, membrane trafficking, immune response	toxic protein aggregation, calcium dysregulation, ER stress, metabolic dysfunction, aberrant synapses, microglia, and astrocyte dysfunction

**Table 2:** Overview of properties of motor neuron subtypes.

Motor Neuron Subtype	Size	Muscle Fiber Type and Metabolism	Muscle Fiber Innervated No.	Vulnerability in ALS	Firing Ability and Contraction	Calcium Handling Properties
Fast-twitch, fast-fatigable (FF)	Large	Glycolytic muscle fibers: MHC type IIb (MYH4), sometimes combined with MHC type IIx (MYH1)	300–2000	Most vulnerable, degenerate early	Low excitability, fast firing, the strongest contractions, and are rapidly fatigued	Lowest ability to buffer calcium
Fast-twitch, fatigue-resistant (FR)	Large	Mixed glycolytic and oxidative energy metabolism: MHC type IIa (MYH2)	Several hundreds	Less vulnerable, degenerate later	Low excitability, fast firing, strong contractions, and less fatigability	Low calcium buffering ability
Slow-twitch, fatigue resistant (S)	Small	Mostly oxidative metabolism: MHC type I (MYH7)	Less than 200	Least vulnerable, degenerate last	Slower firing velocity, relatively weak contractions, resistant to fatigue during prolonged stimulation	Highest calcium buffering ability

**Table 3:** Sources of toxic hyperexcitability in ALS.

Source of Hyperexcitability	Effects
AMPA receptor dysregulation	More AMPA receptors, more AMPA receptors trafficked to synapse, higher GluA1 expression, higher permeability to calcium, higher AMPA signal amplitude
NMDA receptor dysregulation	Increased expression of extrasynaptic NMDA receptors, increased vulnerability to cell death pathway activation
Depolarized resting membrane potential	Increased vulnerability to hyperexcitability in response to synaptic input, increased vulnerability to excitotoxicity
Aberrant synapse formation, structure, and function	Increased excitatory synapse number, increased synaptic vesicle release, and increased vulnerability to hyperexcitability
Reduction in calcium-binding protein levels	Less calcium buffering ability, increased vulnerability to hyperexcitability
GABA receptor dysfunction	Less calcium buffering ability, increased vulnerability

**Table 4:** Instances of hyperexcitability being neuroprotective in ALS and ALS models.

Finding	Effect
S-type but not F-type motor neurons are hyperexcitable	S-type motor neurons resistant to degeneration in ALS; F-type neurons are vulnerable and degenerate first
Trigeminal motor neurons show altered firing thresholds but not oculomotor neurons	Trigeminal motor neurons are vulnerable to degeneration, and oculomotor neurons are resistant
Interneurons are less excitable and have an altered dendritic structure	Interneurons degenerate in early disease stages
Elevated neural calcium levels drive activation of calcium-dependent proteins and proteases	Prevention and reduction in the expression of toxic proteins

**Table 5:** Comparison of the advantages and limitations of in vitro human cell models and in vivo rodent models.

Model	Advantages	Limitations
in vitro human cell models	Human-specific context, avoids species-specific protein differences, can generate precision-targeted therapies for individual patients, high-throughput capacity, tightly controlled environment	Simplified model in a dish compared to a living organism, loss of some complex interactions between different cells and system-system interactions, morphological differences for cultured cells vs. in vivo, particularly for neurons, where structure is strongly shaped by synaptic input
In vivo rodent models	Allow for studying disease in a living organism context, captures complex interactions between different organs and systems, enables long-term longitudinal studies, can utilize behavioral analysis, can assess treatment effects and potential side-effects in a living organism	Species differences in protein expression, a lab-generated rodent model of a disease may not totally reflect the real human disease, and often have a lower throughput capacity

**Table 6:** Potential calcium related treatments targets for ALS.

Cell Type	Target	Therapeutic Mechanism
Motor neurons	Calcium channels	Use agonists in early stages to activate compensatory mechanisms and channel-blocking drugs in later stages to prevent calcium excitotoxicity
	Calcium-binding proteins	Upregulate calcium-binding proteins like parvalbumin and calbindin to prevent calcium excitotoxicity
	AMPA receptors	Modulate the permeability of AMPA receptors through targeting the editing of the GluA2 subunit via ADAR2
	Calcineurin	Upregulate calcineurin activity, reduce toxic phosphorylated TDP-43 accumulation
	ER and mitochondria	Target abnormal calcium management to restore function
Interneurons	Calcium channels	Use calcium channel agonists to stimulate increased firing and prevent early-stage degeneration
	ER and mitochondria	Target abnormal calcium management to restore function
Glial cells	Calcium-activated potassium channels	Inhibit the channel to rescue normal microglia function
	Calcium channels	inhibit channels, prevent elevated calcium levels, and glial malfunction

## Data Availability

No new data were created or analyzed in this study.

## References

[1] Arnold F.J., Putka A.F., Raychaudhuri U., Hsu S., Bedlack R.S., Bennett C.L., La Spada A.R. (2024). Revisiting Glutamate Excitotoxicity in Amyotrophic Lateral Sclerosis and Age-Related Neurodegeneration. Int. J. Mol. Sci..

[2] Brotman R.G., Moreno-Escobar M.C., Joseph J., Munakomi S., Pawar G. (2025). Amyotrophic Lateral Sclerosis. StatPearls.

[3] Talbott E.O., Malek A.M., Lacomis D. (2016). The Epidemiology of Amyotrophic Lateral Sclerosis. Handb. Clin. Neurol..

[4] Ingre C., Roos P.M., Piehl F., Kamel F., Fang F. (2015). Risk Factors for Amyotrophic Lateral Sclerosis. Clin. Epidemiol..

[5] Arnold W.D., Castoro R., Saxena S. (2025). Innovations In Physical Medicine and Rehabilitation: Advances in the Diagnosis, Treatment, and Care of Amyotrophic Lateral Sclerosis. Mo. Med..

[6] Lee J.W., Kang S.-W., Choi W.A. (2021). Clinical Course of Amyotrophic Lateral Sclerosis According to Initial Symptoms: An Analysis of 500 Cases. Yonsei Med. J..

[7] Chen L., Chen G., Zhang M., Zhang X. (2024). Modeling Sporadic Juvenile ALS in iPSC-Derived Motor Neurons Explores the Pathogenesis of FUSR503fs Mutation. Front. Cell. Neurosci..

[8] Quinn C., Elman L. (2020). Amyotrophic Lateral Sclerosis and Other Motor Neuron Diseases. Contin. Lifelong Learn. Neurol..

[9] Morgan S., Orrell R.W. (2016). Pathogenesis of Amyotrophic Lateral Sclerosis. Br. Med. Bull..

[10] Rowland L.P., Shneider N.A. (2001). Amyotrophic Lateral Sclerosis. N. Engl. J. Med..

[11] Brown R.H., Al-Chalabi A. (2017). Amyotrophic Lateral Sclerosis. N. Engl. J. Med..

[12] Vanderhaeghe S., Prerad J., Tharkeshwar A.K., Goethals E., Vints K., Beckers J., Scheveneels W., Debroux E., Princen K., Van Damme P. (2024). A Pathogenic Mutation in the ALS/FTD Gene VCP Induces Mitochondrial Hypermetabolism by Modulating the Permeability Transition Pore. Acta Neuropathol. Commun..

[13] Grad L.I., Rouleau G.A., Ravits J., Cashman N.R. (2017). Clinical Spectrum of Amyotrophic Lateral Sclerosis (ALS). Cold Spring Harb. Perspect. Med..

[14] Leal S.S., Gomes C.M. (2015). Calcium Dysregulation Links ALS Defective Proteins and Motor Neuron Selective Vulnerability. Front. Cell. Neurosci..

[15] Bond S., Saxena S., Sierra-Delgado J.A. (2025). Microglia in ALS: Insights into Mechanisms and Therapeutic Potential. Cells.

[16] Chia R., Chiò A., Traynor B.J. (2018). Novel Genes Associated with Amyotrophic Lateral Sclerosis: Diagnostic and Clinical Implications. Lancet Neurol..

[17] Renton A.E., Chiò A., Traynor B.J. (2014). State of Play in Amyotrophic Lateral Sclerosis Genetics. Nat. Neurosci..

[18] Nijs M., Van Damme P. (2024). The Genetics of Amyotrophic Lateral Sclerosis. Curr. Opin. Neurol..

[19] Goutman S.A., Hardiman O., Al-Chalabi A., Chió A., Savelieff M.G., Kiernan M.C., Feldman E.L. (2022). Emerging Insights into the Complex Genetics and Pathophysiology of Amyotrophic Lateral Sclerosis. Lancet Neurol..

[20] Bagyinszky E., Hulme J., An S.S.A. (2023). Studies of Genetic and Proteomic Risk Factors of Amyotrophic Lateral Sclerosis Inspire Biomarker Development and Gene Therapy. Cells.

[21] Kim G., Gautier O., Tassoni-Tsuchida E., Ma X.R., Gitler A.D. (2020). ALS Genetics: Gains, Losses, and Implications for Future Therapies. Neuron.

[22] Chiò A., Traynor B.J., Lombardo F., Fimognari M., Calvo A., Ghiglione P., Mutani R., Restagno G. (2008). Prevalence of SOD1 Mutations in the Italian ALS Population. Neurology.

[23] Neumann M., Sampathu D.M., Kwong L.K., Truax A.C., Micsenyi M.C., Chou T.T., Bruce J., Schuck T., Grossman M., Clark C.M. (2006). Ubiquitinated TDP-43 in Frontotemporal Lobar Degeneration and Amyotrophic Lateral Sclerosis. Science.

[24] Kabashi E., Bercier V., Lissouba A., Liao M., Brustein E., Rouleau G.A., Drapeau P. (2011). FUS and TARDBP but Not SOD1 Interact in Genetic Models of Amyotrophic Lateral Sclerosis. PLoS Genet..

[25] Johnson J.O., Mandrioli J., Benatar M., Abramzon Y., Van Deerlin V.M., Trojanowski J.Q., Gibbs J.R., Brunetti M., Gronka S., Wuu J. (2010). Exome Sequencing Reveals VCP Mutations as a Cause of Familial ALS. Neuron.

[26] Pilotto F., Smeele P.H., Scheidegger O., Diab R., Schobesberger M., Sierra-Delgado J.A., Saxena S. (2025). Kaempferol Enhances ER-Mitochondria Coupling and Protects Motor Neurons from Mitochondrial Dysfunction and ER Stress in C9ORF72-ALS. Acta Neuropathol. Commun..

[27] Masrori P., Van Damme P. (2020). Amyotrophic Lateral Sclerosis: A Clinical Review. Eur. J. Neurol..

[28] DeJesus-Hernandez M., Mackenzie I.R., Boeve B.F., Boxer A.L., Baker M., Rutherford N.J., Nicholson A.M., Finch N.A., Flynn H., Adamson J. (2011). Expanded GGGGCC Hexanucleotide Repeat in Noncoding Region of C9ORF72 Causes Chromosome 9p-Linked FTD and ALS. Neuron.

[29] Renton A.E., Majounie E., Waite A., Simón-Sánchez J., Rollinson S., Gibbs J.R., Schymick J.C., Laaksovirta H., van Swieten J.C., Myllykangas L. (2011). A Hexanucleotide Repeat Expansion in C9ORF72 Is the Cause of Chromosome 9p21-Linked ALS-FTD. Neuron.

[30] Jiang L., Zhang T., Lu K., Qi S. (2022). The Progress in C9orf72 Research: ALS/FTD Pathogenesis, Functions and Structure. Small GTPases 13.

[31] Smeyers J., Banchi E.-G., Latouche M. (2021). C9ORF72: What It Is, What It Does, and Why It Matters. Front. Cell. Neurosci..

[32] Bozzoni V., Pansarasa O., Diamanti L., Nosari G., Cereda C., Ceroni M. (2016). Amyotrophic Lateral Sclerosis and Environmental Factors. Funct. Neurol..

[33] Morahan J.M., Yu B., Trent R.J., Pamphlett R. (2007). Genetic Susceptibility to Environmental Toxicants in ALS. Am. J. Med. Genet. Part B Neuropsychiatr. Genet. Off. Publ. Int. Soc. Psychiatr. Genet..

[34] Verma A., Araki T. (2021). Clinical Manifestation and Management of Amyotrophic Lateral Sclerosis. Amyotrophic Lateral Sclerosis.

[35] de Carvalho M., Swash M., Pinto S. (2019). Diaphragmatic Neurophysiology and Respiratory Markers in ALS. Front. Neurol..

[36] Abramzon Y.A., Fratta P., Traynor B.J., Chia R. (2020). The Overlapping Genetics of Amyotrophic Lateral Sclerosis and Frontotemporal Dementia. Front. Neurosci..

[37] Burrell J.R., Kiernan M.C., Vucic S., Hodges J.R. (2011). Motor Neuron Dysfunction in Frontotemporal Dementia. Brain J. Neurol..

[38] Hur S.K., Hunter M., Dominique M.A., Farag M., Cotton-Samuel D., Khan T., Trojanowski J.Q., Spiller K.J., Lee V.M.-Y. (2022). Slow Motor Neurons Resist Pathological TDP-43 and Mediate Motor Recovery in the rNLS8 Model of Amyotrophic Lateral Sclerosis. Acta Neuropathol. Commun..

[39] Ovsepian S.V., O’Leary V.B., Martinez S. (2024). Selective Vulnerability of Motor Neuron Types and Functional Groups to Degeneration in Amyotrophic Lateral Sclerosis: Review of the Neurobiological Mechanisms and Functional Correlates. Brain Struct. Funct..

[40] Díaz-García C.M., Meyer D.J., Nathwani N., Rahman M., Martínez-François J.R., Yellen G. (2021). The Distinct Roles of Calcium in Rapid Control of Neuronal Glycolysis and the Tricarboxylic Acid Cycle. eLife.

[41] Südhof T.C. (2012). Calcium Control of Neurotransmitter Release. Cold Spring Harb. Perspect. Biol..

[42] Gleichmann M., Mattson M.P. (2011). Neuronal Calcium Homeostasis and Dysregulation. Antioxid. Redox Signal..

[43] Brini M., Calì T., Ottolini D., Carafoli E. (2014). Neuronal Calcium Signaling: Function and Dysfunction. Cell. Mol. Life Sci. CMLS.

[44] Zündorf G., Reiser G. (2011). Calcium Dysregulation and Homeostasis of Neural Calcium in the Molecular Mechanisms of Neurodegenerative Diseases Provide Multiple Targets for Neuroprotection. Antioxid. Redox Signal..

[45] Matuz-Mares D., González-Andrade M., Araiza-Villanueva M.G., Vilchis-Landeros M.M., Vázquez-Meza H. (2022). Mitochondrial Calcium: Effects of Its Imbalance in Disease. Antioxidants.

[46] Dafinca R., Barbagallo P., Farrimond L., Candalija A., Scaber J., Ababneh N.A., Sathyaprakash C., Vowles J., Cowley S.A., Talbot K. (2020). Impairment of Mitochondrial Calcium Buffering Links Mutations in C9ORF72 and TARDBP in iPS-Derived Motor Neurons from Patients with ALS/FTD. Stem Cell Rep..

[47] Bursch F., Kalmbach N., Naujock M., Staege S., Eggenschwiler R., Abo-Rady M., Japtok J., Guo W., Hensel N., Reinhardt P. (2019). Altered Calcium Dynamics and Glutamate Receptor Properties in iPSC-Derived Motor Neurons from ALS Patients with C9orf72, FUS, SOD1 or TDP43 Mutations. Hum. Mol. Genet..

[48] Selvaraj B.T., Livesey M.R., Zhao C., Gregory J.M., James O.T., Cleary E.M., Chouhan A.K., Gane A.B., Perkins E.M., Dando O. (2018). C9ORF72 Repeat Expansion Causes Vulnerability of Motor Neurons to Ca^2+^-Permeable AMPA Receptor-Mediated Excitotoxicity. Nat. Commun..

[49] Kawahara Y., Ito K., Sun H., Aizawa H., Kanazawa I., Kwak S. (2004). Glutamate Receptors: RNA Editing and Death of Motor Neurons. Nature.

[50] Takuma H., Kwak S., Yoshizawa T., Kanazawa I. (1999). Reduction of GluR2 RNA Editing, a Molecular Change That Increases Calcium Influx through AMPA Receptors, Selective in the Spinal Ventral Gray of Patients with Amyotrophic Lateral Sclerosis. Ann. Neurol..

[51] Guo C., Ma Y.-Y. (2021). Calcium Permeable-AMPA Receptors and Excitotoxicity in Neurological Disorders. Front. Neural Circuits.

[52] Hideyama T., Yamashita T., Suzuki T., Tsuji S., Higuchi M., Seeburg P.H., Takahashi R., Misawa H., Kwak S. (2010). Induced Loss of ADAR2 Engenders Slow Death of Motor Neurons from Q/R Site-Unedited GluR2. J. Neurosci..

[53] Huber N., Hoffmann D., Giniatullina R., Rostalski H., Leskelä S., Takalo M., Natunen T., Solje E., Remes A.M., Giniatullin R. (2022). C9orf72 Hexanucleotide Repeat Expansion Leads to Altered Neuronal and Dendritic Spine Morphology and Synaptic Dysfunction. Neurobiol. Dis..

[54] Chikuchi R., Kato Y., Tomatsu A., Nishisaki S., Kawakami Y., Yoshimura T., Li J., Iguchi Y., Onodera K., Hashimoto R. (2026). The TDP-43I383V Heterozygous Mutation Results in Increased TDP-43 Expression and Altered Neuronal Activity in ALS Patient-Derived iPSC Motor Neurons. Neurosci. Res..

[55] Grosskreutz J., Van Den Bosch L., Keller B.U. (2010). Calcium Dysregulation in Amyotrophic Lateral Sclerosis. Cell Calcium.

[56] Vanselow B.K., Keller B.U. (2000). Calcium Dynamics and Buffering in Oculomotor Neurones from Mouse That Are Particularly Resistant during Amyotrophic Lateral Sclerosis (ALS)-Related Motoneurone Disease. J. Physiol..

[57] Palecek J., Lips M.B., Keller B.U. (1999). Calcium Dynamics and Buffering in Motoneurones of the Mouse Spinal Cord. J. Physiol..

[58] Amalyan S., Tamboli S., Lazarevich I., Topolnik D., Bouman L.H., Topolnik L. (2022). Enhanced Motor Cortex Output and Disinhibition in Asymptomatic Female Mice with *C9orf72* Genetic Expansion. Cell Rep..

[59] Jaiswal M.K. (2013). Calcium, Mitochondria, and the Pathogenesis of ALS: The Good, the Bad, and the Ugly. Front. Cell. Neurosci..

[60] Alexianu M.E., Ho B.-K., Mohamed A.H., La Bella V., Smith R.G., Appel S.H. (1994). The Role of Calcium-Binding Proteins in Selective Motoneuron Vulnerability in Amyotrophic Lateral Sclerosis. Ann. Neurol..

[61] Sun D., Amiri M., Meng Q., Unnithan R.R., French C. (2024). Calcium Signalling in Neurological Disorders, with Insights from Miniature Fluorescence Microscopy. Cells.

[62] Tran L.T., Gentil B.J., Sullivan K.E., Durham H.D. (2014). The Voltage-Gated Calcium Channel Blocker Lomerizine Is Neuroprotective in Motor Neurons Expressing Mutant SOD1, but Not TDP-43. J. Neurochem..

[63] Patten S.A., Aggad D., Martinez J., Tremblay E., Petrillo J., Armstrong G.A., La Fontaine A., Maios C., Liao M., Ciura S. (2017). Neuroleptics as Therapeutic Compounds Stabilizing Neuromuscular Transmission in Amyotrophic Lateral Sclerosis. JCI Insight.

[64] Pozzi S., Thammisetty S.S., Julien J.-P. (2018). Chronic Administration of Pimozide Fails to Attenuate Motor and Pathological Deficits in Two Mouse Models of Amyotrophic Lateral Sclerosis. Neurother. J. Am. Soc. Exp. Neurother..

[65] Shum C., Hedges E.C., Allison J., Lee Y., Arias N., Cocks G., Chandran S., Ruepp M.-D., Shaw C.E., Nishimura A.L. (2024). Mutations in FUS Lead to Synaptic Dysregulation in ALS-iPSC Derived Neurons. Stem Cell Rep..

[66] Meszlényi V., Patai R., Polgár T.F., Nógrádi B., Körmöczy L., Kristóf R., Spisák K., Tripolszki K., Széll M., Obál I. (2020). Passive Transfer of Sera from ALS Patients with Identified Mutations Evokes an Increased Synaptic Vesicle Number and Elevation of Calcium Levels in Motor Axon Terminals, Similar to Sera from Sporadic Patients. Int. J. Mol. Sci..

[67] Foerster B.R., Callaghan B.C., Petrou M., Edden R.a.E., Chenevert T.L., Feldman E.L. (2012). Decreased Motor Cortex γ-Aminobutyric Acid in Amyotrophic Lateral Sclerosis. Neurology.

[68] Weerasekera A., Peeters R., Sima D., Dresselaers T., Sunaert S., De Vocht J., Claeys K., Van Huffel S., Van Damme P., Himmelreich U. (2019). Motor Cortex Metabolite Alterations in Amyotrophic Lateral Sclerosis Assessed in Vivo Using Edited and Non-Edited Magnetic Resonance Spectroscopy. Brain Res..

[69] Hinchcliffe M., Smith A. (2017). Riluzole: Real-World Evidence Supports Significant Extension of Median Survival Times in Patients with Amyotrophic Lateral Sclerosis. Degener. Neurol. Neuromuscul. Dis..

[70] Leroy F., Lamotte d’Incamps B., Imhoff-Manuel R.D., Zytnicki D. (2014). Early Intrinsic Hyperexcitability Does Not Contribute to Motoneuron Degeneration in Amyotrophic Lateral Sclerosis. eLife.

[71] Venugopal S., Hsiao C.-F., Sonoda T., Wiedau-Pazos M., Chandler S.H. (2015). Homeostatic Dysregulation in Membrane Properties of Masticatory Motoneurons Compared with Oculomotor Neurons in a Mouse Model for Amyotrophic Lateral Sclerosis. J. Neurosci..

[72] Michalak M. (2023). Calreticulin: Endoplasmic Reticulum Ca^2+^ Gatekeeper. J. Cell. Mol. Med..

[73] Clark R.M., Blizzard C.A., Young K.M., King A.E., Dickson T.C. (2017). Calretinin and Neuropeptide Y Interneurons Are Differentially Altered in the Motor Cortex of the SOD1G93A Mouse Model of ALS. Sci. Rep..

[74] Petri S., Krampfl K., Hashemi F., Grothe C., Hori A., Dengler R., Bufler J. (2003). Distribution of GABAA Receptor mRNA in the Motor Cortex of ALS Patients. J. Neuropathol. Exp. Neurol..

[75] Mazurie Z., Branchereau P., Cattaert D., Henkous N., Savona-Baron C., Vouimba R.-M. (2024). Acute Stress Differently Modulates Interneurons Excitability and Synaptic Plasticity in the Primary Motor Cortex of Wild-Type and SOD1G93A Mouse Model of ALS. J. Physiol..

[76] Montañana-Rosell R., Selvan R., Hernández-Varas P., Kaminski J.M., Sidhu S.K., Ahlmark D.B., Kiehn O., Allodi I. (2024). Spinal Inhibitory Neurons Degenerate before Motor Neurons and Excitatory Neurons in a Mouse Model of ALS. Sci. Adv..

[77] Nijssen J., Comley L.H., Hedlund E. (2017). Motor Neuron Vulnerability and Resistance in Amyotrophic Lateral Sclerosis. Acta Neuropathol..

[78] Schweingruber C., Hedlund E. (2022). The Cell Autonomous and Non-Cell Autonomous Aspects of Neuronal Vulnerability and Resilience in Amyotrophic Lateral Sclerosis. Biology.

[79] De Marco G., Lomartire A., Manera U., Canosa A., Grassano M., Casale F., Fuda G., Salamone P., Rinaudo M.T., Colombatto S. (2022). Effects of Intracellular Calcium Accumulation on Proteins Encoded by the Major Genes Underlying Amyotrophic Lateral Sclerosis. Sci. Rep..

[80] Park J.H., Chung C.G., Park S.S., Lee D., Kim K.M., Jeong Y., Kim E.S., Cho J.H., Jeon Y.-M., Shen C.-K.J. (2020). Cytosolic Calcium Regulates Cytoplasmic Accumulation of TDP-43 through Calpain-A and Importin A3. eLife.

[81] Waldherr S.M., Eck R.J., Hincks J.C., Currey H.N., Goldberg M., McMillan P.J., Saxton A.D., Hulsey-Vincent H.J., Latimer C.S., Kraemer B.C. (2026). Calcineurin Depletion Coincides with Phosphorylated TDP-43 Deposition in a Mouse Model of ALS/FTLD-TDP. Acta Neuropathol. Commun..

[82] Chen L., Song M., Yao C. (2022). Calcineurin in Development and Disease. Genes Dis..

[83] Liachko N.F., Saxton A.D., McMillan P.J., Strovas T.J., Currey H.N., Taylor L.M., Wheeler J.M., Oblak A.L., Ghetti B., Montine T.J. (2016). The Phosphatase Calcineurin Regulates Pathological TDP-43 Phosphorylation. Acta Neuropathol..

[84] Armstrong G.A.B., Drapeau P. (2013). Calcium Channel Agonists Protect against Neuromuscular Dysfunction in a Genetic Model of TDP-43 Mutation in ALS. J. Neurosci..

[85] Saxena S., Roselli F., Singh K., Leptien K., Julien J.-P., Gros-Louis F., Caroni P. (2013). Neuroprotection through Excitability and mTOR Required in ALS Motoneurons to Delay Disease and Extend Survival. Neuron.

[86] Wate R., Ito H., Zhang J.H., Ohnishi S., Nakano S., Kusaka H. (2005). Expression of an Endoplasmic Reticulum-Resident Chaperone, Glucose-Regulated Stress Protein 78, in the Spinal Cord of a Mouse Model of Amyotrophic Lateral Sclerosis. Acta Neuropathol..

[87] Kikuchi H., Almer G., Yamashita S., Guégan C., Nagai M., Xu Z., Sosunov A.A., McKhann G.M., Przedborski S. (2006). Spinal Cord Endoplasmic Reticulum Stress Associated with a Microsomal Accumulation of Mutant Superoxide Dismutase-1 in an ALS Model. Proc. Natl. Acad. Sci. USA.

[88] Zhao C., Liao Y., Rahaman A., Kumar V. (2022). Towards Understanding the Relationship Between ER Stress and Unfolded Protein Response in Amyotrophic Lateral Sclerosis. Front. Aging Neurosci..

[89] Bernard-Marissal N., Moumen A., Sunyach C., Pellegrino C., Dudley K., Henderson C.E., Raoul C., Pettmann B. (2012). Reduced Calreticulin Levels Link Endoplasmic Reticulum Stress and Fas-Triggered Cell Death in Motoneurons Vulnerable to ALS. J. Neurosci..

[90] Saxena S., Cabuy E., Caroni P. (2009). A Role for Motoneuron Subtype-Selective ER Stress in Disease Manifestations of FALS Mice. Nat. Neurosci..

[91] Tadic V., Prell T., Lautenschlaeger J., Grosskreutz J. (2014). The ER Mitochondria Calcium Cycle and ER Stress Response as Therapeutic Targets in Amyotrophic Lateral Sclerosis. Front. Cell. Neurosci..

[92] De Lorenzo F., Lüningschrör P., Nam J., Beckett L., Pilotto F., Galli E., Lindholm P., Rüdt von Collenberg C., Mungwa S.T., Jablonka S. (2023). CDNF Rescues Motor Neurons in Models of Amyotrophic Lateral Sclerosis by Targeting Endoplasmic Reticulum Stress. Brain.

[93] Moss K.R., Saxena S. (2025). Schwann Cells in Neuromuscular Disorders: A Spotlight on Amyotrophic Lateral Sclerosis. Cells.

[94] Verma M., Wills Z., Chu C.T. (2018). Excitatory Dendritic Mitochondrial Calcium Toxicity: Implications for Parkinson’s and Other Neurodegenerative Diseases. Front. Neurosci..

[95] Lautenschläger J., Prell T., Ruhmer J., Weidemann L., Witte O.W., Grosskreutz J. (2013). Overexpression of Human Mutated G93A SOD1 Changes Dynamics of the ER Mitochondria Calcium Cycle Specifically in Mouse Embryonic Motor Neurons. Exp. Neurol..

[96] Taylor C.W., Tovey S.C. (2010). IP3 Receptors: Toward Understanding Their Activation. Cold Spring Harb. Perspect. Biol..

[97] Wuytack F., Raeymaekers L., Missiaen L. (2002). Molecular Physiology of the SERCA and SPCA Pumps. Cell Calcium.

[98] Gunter T.E., Sheu S.-S. (2009). Characteristics and Possible Functions of Mitochondrial Ca^2+^ Transport Mechanisms. Biochim. Biophys. Acta BBA—Bioenerg..

[99] Pivovarova N.B., Andrews S.B. (2010). Calcium-Dependent Mitochondrial Function and Dysfunction in Neurons. FEBS J..

[100] Pilotto F., Toth T.D., Bond S., Schmitz A., Diab R., Tenlep S.Y.N., Mooney B., Erni S., Schobesberger M., Scheidegger O. (2026). Engineered GM1 Intersects Between Mitochondrial and Synaptic Pathways to Ameliorate ALS Pathology. Adv. Sci..

[101] Jaiswal M.K., Zech W.-D., Goos M., Leutbecher C., Ferri A., Zippelius A., Carrì M.T., Nau R., Keller B.U. (2009). Impairment of Mitochondrial Calcium Handling in a mtSOD1 Cell Culture Model of Motoneuron Disease. BMC Neurosci..

[102] Božič J., Motaln H., Janež A.P., Markič L., Tripathi P., Yamoah A., Aronica E., Lee Y.-B., Heilig R., Fischer R. (2022). Interactome Screening of *C9orf72* Dipeptide Repeats Reveals VCP Sequestration and Functional Impairment by polyGA. Brain.

[103] Pilotto F., Schmitz A., Maharjan N., Diab R., Odriozola A., Tripathi P., Yamoah A., Scheidegger O., Oestmann A., Dennys C.N. (2022). PolyGA Targets the ER Stress-Adaptive Response by Impairing GRP75 Function at the MAM in C9ORF72-ALS/FTD. Acta Neuropathol..

[104] Raoul C., Estévez A.G., Nishimune H., Cleveland D.W., deLapeyrière O., Henderson C.E., Haase G., Pettmann B. (2002). Motoneuron Death Triggered by a Specific Pathway Downstream of Fas: Potentiation by ALS-Linked SOD1 Mutations. Neuron.

[105] Kawamata H., Manfredi G. (2010). Mitochondrial Dysfunction and Intracellular Calcium Dysregulation in ALS. Mech. Ageing Dev..

[106] Gupta R., Santiago E., Verma A., Rao R. (2025). Calcium Dysregulation in Amyotrophic Lateral Sclerosis. Physiology.

[107] Ruegsegger C., Maharjan N., Goswami A., Filézac de L’Etang A., Weis J., Troost D., Heller M., Gut H., Saxena S. (2016). Aberrant Association of Misfolded SOD1 with Na^+/^K^+^ATPase-α3 Impairs Its Activity and Contributes to Motor Neuron Vulnerability in ALS. Acta Neuropathol..

[108] Nahm M., Lim S.M., Kim Y.-E., Park J., Noh M.-Y., Lee S., Roh J.E., Hwang S.-M., Park C.-K., Kim Y.H. (2020). ANXA11 Mutations in ALS Cause Dysregulation of Calcium Homeostasis and Stress Granule Dynamics. Sci. Transl. Med..

[109] Xie M., Pallegar P.N., Parusel S., Nguyen A.T., Wu L.-J. (2023). Regulation of Cortical Hyperexcitability in Amyotrophic Lateral Sclerosis: Focusing on Glial Mechanisms. Mol. Neurodegener..

[110] Isbrandt D. (2017). A Mechanistic Link between Glia and Neuronal Excitability in Acute Neuroinflammation. J. Physiol..

[111] Pascual O., Ben Achour S., Rostaing P., Triller A., Bessis A. (2012). Microglia Activation Triggers Astrocyte-Mediated Modulation of Excitatory Neurotransmission. Proc. Natl. Acad. Sci. USA.

[112] Chen T., Lennon V.A., Liu Y.U., Bosco D.B., Li Y., Yi M.-H., Zhu J., Wei S., Wu L.-J. (2020). Astrocyte-Microglia Interaction Drives Evolving Neuromyelitis Optica Lesion. J. Clin. Investig..

[113] Cocozza G., di Castro M.A., Carbonari L., Grimaldi A., Antonangeli F., Garofalo S., Porzia A., Madonna M., Mainiero F., Santoni A. (2018). Ca^2+^-Activated K^+^ Channels Modulate Microglia Affecting Motor Neuron Survival in hSOD1G93A Mice. Brain. Behav. Immun..

[114] Cocozza G., Garofalo S., Morotti M., Chece G., Grimaldi A., Lecce M., Scavizzi F., Menghini R., Casagrande V., Federici M. (2021). The Feeding Behaviour of Amyotrophic Lateral Sclerosis Mouse Models Is Modulated by the Ca^2+^-activated KCa3.1 Channels. Br. J. Pharmacol..

[115] Kerk S.Y., Bai Y., Smith J., Lalgudi P., Hunt C., Kuno J., Nuara J., Yang T., Lanza K., Chan N. (2022). Homozygous ALS-Linked FUS P525L Mutations Cell- Autonomously Perturb Transcriptome Profile and Chemoreceptor Signaling in Human iPSC Microglia. Stem Cell Rep..

[116] Poon H.F., Hensley K., Thongboonkerd V., Merchant M.L., Lynn B.C., Pierce W.M., Klein J.B., Calabrese V., Butterfield D.A. (2005). Redox Proteomics Analysis of Oxidatively Modified Proteins in G93A-SOD1 Transgenic Mice—A Model of Familial Amyotrophic Lateral Sclerosis. Free Radic. Biol. Med..

[117] Cassina P., Cassina A., Pehar M., Castellanos R., Gandelman M., de León A., Robinson K.M., Mason R.P., Beckman J.S., Barbeito L. (2008). Mitochondrial Dysfunction in SOD1G93A-Bearing Astrocytes Promotes Motor Neuron Degeneration: Prevention by Mitochondrial-Targeted Antioxidants. J. Neurosci..

[118] Kawamata H., Ng S.K., Diaz N., Burstein S., Morel L., Osgood A., Sider B., Higashimori H., Haydon P.G., Manfredi G. (2014). Abnormal Intracellular Calcium Signaling and SNARE-Dependent Exocytosis Contributes to SOD1G93A Astrocyte-Mediated Toxicity in Amyotrophic Lateral Sclerosis. J. Neurosci. Off. J. Soc. Neurosci..

[119] Milošević M., Bataveljić D., Nikolić L., Bijelić D., Andjus P. (2016). The Effect of Amyotrophic Lateral Sclerosis-Linked Exogenous SOD1-G93A on Electrophysiological Properties and Intracellular Calcium in Cultured Rat Astrocytes. Amyotroph. Lateral Scler. Front. Degener..

[120] Norante R.P., Peggion C., Rossi D., Martorana F., De Mario A., Lia A., Massimino M.L., Bertoli A. (2019). ALS-Associated SOD1(G93A) Decreases SERCA Pump Levels and Increases Store-Operated Ca^2+^ Entry in Primary Spinal Cord Astrocytes from a Transgenic Mouse Model. Int. J. Mol. Sci..

[121] Velebit J., Horvat A., Smolič T., Prpar Mihevc S., Rogelj B., Zorec R., Vardjan N. (2020). Astrocytes with TDP-43 Inclusions Exhibit Reduced Noradrenergic cAMP and Ca2+ Signaling and Dysregulated Cell Metabolism. Sci. Rep..

[122] Fritz E., Izaurieta P., Weiss A., Mir F.R., Rojas P., Gonzalez D., Rojas F., Brown R.H., Madrid R., van Zundert B. (2013). Mutant SOD1-Expressing Astrocytes Release Toxic Factors That Trigger Motoneuron Death by Inducing Hyperexcitability. J. Neurophysiol..

[123] Gunes Z.I., Kan V.W.Y., Ye X., Liebscher S. (2020). Exciting Complexity: The Role of Motor Circuit Elements in ALS Pathophysiology. Front. Neurosci..

[124] Martorana F., Brambilla L., Valori C.F., Bergamaschi C., Roncoroni C., Aronica E., Volterra A., Bezzi P., Rossi D. (2012). The BH4 Domain of Bcl-X(L) Rescues Astrocyte Degeneration in Amyotrophic Lateral Sclerosis by Modulating Intracellular Calcium Signals. Hum. Mol. Genet..

[125] McCauley M.E., Baloh R.H. (2019). Inflammation in ALS/FTD Pathogenesis. Acta Neuropathol..

[126] Jong Huat T., Camats-Perna J., Newcombe E.A., Onraet T., Campbell D., Sucic J.T., Martini A., Forner S., Mirzaei M., Poon W. (2024). The Impact of Astrocytic NF-κB on Healthy and Alzheimer’s Disease Brains. Sci. Rep..

[127] Lee H.-K., Barbarosie M., Kameyama K., Bear M.F., Huganir R.L. (2000). Regulation of Distinct AMPA Receptor Phosphorylation Sites during Bidirectional Synaptic Plasticity. Nature.

[128] Douthwaite C., Tietje C., Ye X., Liebscher S. (2024). Probing Cerebellar Circuit Dysfunction in Rodent Models of Spinocerebellar Ataxia by Means of in Vivo Two-Photon Calcium Imaging. STAR Protoc..

[129] Croci D., Zomer A., Kowal J., Joyce J.A. (2023). Cranial Imaging Window Implantation Technique for Longitudinal Multimodal Imaging of the Brain Environment in Live Mice. STAR Protoc..

[130] Denk W., Delaney K.R., Gelperin A., Kleinfeld D., Strowbridge B.W., Tank D.W., Yuste R. (1994). Anatomical and Functional Imaging of Neurons Using 2-Photon Laser Scanning Microscopy. J. Neurosci. Methods.

[131] Svoboda K., Denk W., Kleinfeld D., Tank D.W. (1997). In Vivo Dendritic Calcium Dynamics in Neocortical Pyramidal Neurons. Nature.

[132] Rodríguez C., Liang Y., Lu R., Ji N. (2018). Three-Photon Fluorescence Microscopy with an Axially Elongated Bessel Focus. Opt. Lett..

[133] Xiao Y., Deng P., Zhao Y., Yang S., Li B. (2023). Three-Photon Excited Fluorescence Imaging in Neuroscience: From Principles to Applications. Front. Neurosci..

[134] Chen B., Huang X., Gou D., Zeng J., Chen G., Pang M., Hu Y., Zhao Z., Zhang Y., Zhou Z. (2018). Rapid Volumetric Imaging with Bessel-Beam Three-Photon Microscopy. Biomed. Opt. Express.

[135] Aharoni D., Khakh B.S., Silva A.J., Golshani P. (2019). All the Light That We Can See: A New Era in Miniaturized Microscopy. Nat. Methods.

[136] Guo C., Blair G.J., Sehgal M., Sangiuliano Jimka F.N., Bellafard A., Silva A.J., Golshani P., Basso M.A., Blair H.T., Aharoni D. (2023). Miniscope-LFOV: A Large-Field-of-View, Single-Cell-Resolution, Miniature Microscope for Wired and Wire-Free Imaging of Neural Dynamics in Freely Behaving Animals. Sci. Adv..

[137] Werner C.T., Williams C.J., Fermelia M.R., Lin D.-T., Li Y. (2019). Circuit Mechanisms of Neurodegenerative Diseases: A New Frontier with Miniature Fluorescence Microscopy. Front. Neurosci..

[138] Liang B., Thapa R., Zhang G., Moffitt C., Zhang Y., Zhang L., Johnston A., Ruby H.P., Barbera G., Wong P.C. (2022). Aberrant Neural Activity in Prefrontal Pyramidal Neurons Lacking TDP-43 Precedes Neuron Loss. Prog. Neurobiol..

[139] Qian L., Liu Y., Chen Y., Wu J. (2025). High-Throughput Two-Photon Volumetric Brain Imaging in Freely Moving Mice. Nat. Commun..

[140] Zhou A., Engelmann S.A., Mihelic S.A., Tomar A., Hassan A.M., Dunn A.K. (2022). Evaluation of Resonant Scanning as a High-Speed Imaging Technique for Two-Photon Imaging of Cortical Vasculature. Biomed. Opt. Express.

[141] Akondi V., Kowalski B., Burns S.A., Dubra A. (2020). Dynamic Distortion in Resonant Galvanometric Optical Scanners. Optica.

[142] Grewe B.F., Voigt F.F., van ’t Hoff M., Helmchen F. (2011). Fast Two-Layer Two-Photon Imaging of Neuronal Cell Populations Using an Electrically Tunable Lens. Biomed. Opt. Express.

[143] Sakaki K.D.R., Coleman P., Toth T.D., Guerrier C., Haas K. Automating Event-Detection of Brain Neuron Synaptic Activity and Action Potential Firing in Vivo Using a Random-Access Multiphoton Laser Scanning Microscope for Real-Time Analysis. Proceedings of the 2018 40th Annual International Conference of the IEEE Engineering in Medicine and Biology Society (EMBC).

[144] Sakaki K.D.R., Podgorski K., Dellazizzo Toth T.A., Coleman P., Haas K. (2020). Comprehensive Imaging of Sensory-Evoked Activity of Entire Neurons Within the Awake Developing Brain Using Ultrafast AOD-Based Random-Access Two-Photon Microscopy. Front. Neural Circuits.

[145] Chen T.-W., Wardill T.J., Sun Y., Pulver S.R., Renninger S.L., Baohan A., Schreiter E.R., Kerr R.A., Orger M.B., Jayaraman V. (2013). Ultrasensitive Fluorescent Proteins for Imaging Neuronal Activity. Nature.

[146] Nakai J., Ohkura M., Imoto K. (2001). A High Signal-to-Noise Ca^2+^ Probe Composed of a Single Green Fluorescent Protein. Nat. Biotechnol..

[147] Zhang Y., Rózsa M., Liang Y., Bushey D., Wei Z., Zheng J., Reep D., Broussard G.J., Tsang A., Tsegaye G. (2023). Fast and Sensitive GCaMP Calcium Indicators for Imaging Neural Populations. Nature.

[148] Dana H., Sun Y., Mohar B., Hulse B.K., Kerlin A.M., Hasseman J.P., Tsegaye G., Tsang A., Wong A., Patel R. (2019). High-Performance Calcium Sensors for Imaging Activity in Neuronal Populations and Microcompartments. Nat. Methods.

[149] Katona G., Szalay G., Maák P., Kaszás A., Veress M., Hillier D., Chiovini B., Vizi E.S., Roska B., Rózsa B. (2012). Fast Two-Photon in Vivo Imaging with Three-Dimensional Random-Access Scanning in Large Tissue Volumes. Nat. Methods.

[150] Sun W., McConnell E., Pare J.-F., Xu Q., Chen M., Peng W., Lovatt D., Han X., Smith Y., Nedergaard M. (2013). Glutamate-Dependent Neuroglial Calcium Signaling Differs Between Young and Adult Brain. Science.

[151] Guerrier C., Dellazizzo Toth T., Galtier N., Haas K. (2023). An Algorithm Based on a Cable-Nernst Planck Model Predicting Synaptic Activity throughout the Dendritic Arbor with Micron Specificity. Neuroinformatics.

[152] Coleman P., Hogg P.W., Toth T.D., Haas K. (2024). PyNeuroTrace—Python Code for Neural Activity Time Series. J. Open Source Softw..

[153] Xie M., Liang Y., Miller A.S., Pallegar P.N., Umpierre A.D., Wang N., Zhang S., Nagaraj N.K., Fogarty Z.C., Ghayal N.B. (2025). Rod-Shaped Microglia Interact with Neuronal Dendrites to Attenuate Cortical Excitability during TDP-43-Related Neurodegeneration. Immunity.

[154] Asakawa K., Handa H., Kawakami K. (2023). Dysregulated TDP-43 Proteostasis Perturbs Excitability of Spinal Motor Neurons during Brainstem-Mediated Fictive Locomotion in Zebrafish. Dev. Growth Differ..

[155] Marvin J.S., Borghuis B.G., Tian L., Cichon J., Harnett M.T., Akerboom J., Gordus A., Renninger S.L., Chen T.-W., Bargmann C.I. (2013). An Optimized Fluorescent Probe for Visualizing Glutamate Neurotransmission. Nat. Methods.

[156] Marvin J.S., Scholl B., Wilson D.E., Podgorski K., Kazemipour A., Müller J.A., Schoch S., Quiroz F.J.U., Rebola N., Bao H. (2018). Stability, Affinity, and Chromatic Variants of the Glutamate Sensor iGluSnFR. Nat. Methods.

[157] Aggarwal A., Liu R., Chen Y., Ralowicz A.J., Bergerson S.J., Tomaska F., Mohar B., Hanson T.L., Hasseman J.P., Reep D. (2023). Glutamate Indicators with Improved Activation Kinetics and Localization for Imaging Synaptic Transmission. Nat. Methods.

[158] Gonzalez K.C., Losonczy A., Negrean A. (2022). Dendritic Excitability and Synaptic Plasticity In Vitro and In Vivo. Neuroscience.

[159] Lavzin M., Rapoport S., Polsky A., Garion L., Schiller J. (2012). Nonlinear Dendritic Processing Determines Angular Tuning of Barrel Cortex Neurons In Vivo. Nature.

[160] Fomin V., Richard P., Hoque M., Li C., Gu Z., Fissore-O’Leary M., Tian B., Prives C., Manley J.L. (2018). The C9ORF72 Gene, Implicated in Amyotrophic Lateral Sclerosis and Frontotemporal Dementia, Encodes a Protein That Functions in Control of Endothelin and Glutamate Signaling. Mol. Cell. Biol..

[161] Deisseroth K. (2011). Optogenetics. Nat. Methods.

[162] Emiliani V., Entcheva E., Hedrich R., Hegemann P., Konrad K.R., Lüscher C., Mahn M., Pan Z.-H., Sims R.R., Vierock J. (2022). Optogenetics for Light Control of Biological Systems. Nat. Rev. Methods Primer.

[163] Asakawa K., Handa H., Kawakami K. (2020). Optogenetic Modulation of TDP-43 Oligomerization Accelerates ALS-Related Pathologies in the Spinal Motor Neurons. Nat. Commun..

[164] Bryson J.B., Kourgiantaki A., Jiang D., Demosthenous A., Greensmith L. (2024). An Optogenetic Cell Therapy to Restore Control of Target Muscles in an Aggressive Mouse Model of Amyotrophic Lateral Sclerosis. eLife.

[165] Northall A., Doehler J., Weber M., Vielhaber S., Schreiber S., Kuehn E. (2023). Layer-Specific Vulnerability Is a Mechanism of Topographic Map Aging. Neurobiol. Aging.

[166] Northall A., Doehler J., Weber M., Tellez I., Petri S., Prudlo J., Vielhaber S., Schreiber S., Kuehn E. (2024). Multimodal Layer Modelling Reveals in Vivo Pathology in Amyotrophic Lateral Sclerosis. Brain.

[167] Ghaderi S., Mohammadi S., Fatehi F. (2024). Calcium Accumulation or Iron Deposition: Delving into the Temporal Sequence of Amyotrophic Lateral Sclerosis Pathophysiology in the Primary Motor Cortex. Ibrain.

